# Dravet Syndrome: A Rare Form of Epilepsy

**DOI:** 10.1155/2024/6710512

**Published:** 2024-07-02

**Authors:** Salwa Al Hosani, Sona Varghese

**Affiliations:** Department of Psychiatry Sheikh Khalifa Medical City, P.O. Box 51900, Abu Dhabi, UAE

## Abstract

Dravet syndrome is a rare and severe form of epilepsy that usually emerges in infancy. It is characterized by diverse seizure patterns, cognitive regression, motor impairments, and behavioral abnormalities. The majority of patients with this condition have mutations involving the voltage-gated sodium channel alpha (I) gene *SCN1A*. We present a detailed account of a two-year-old child with a history of recurrent seizures since the age of 4 months. Genetic testing was performed which revealed a heterozygous pathogenic variant, confirming the diagnosis. The patient was managed successfully by a multidisciplinary approach involving neurologists, developmental specialists, and physical therapists.

## 1. Introduction

Dravet syndromes (DSs), one of the rare intractable epileptic encephalopathies occurring in early childhood, are characterized by diverse seizure patterns, cognitive regression, motor impairments, and behavioral abnormalities [1]. Dravet syndrome was described in 1978 by Dravet (1978) under the name of severe myoclonic epilepsy in infancy (SMEI) but changed to Dravet syndrome in 1989 as the myoclonic component of epilepsy was not always present [2]. Most patients with Dravet syndrome have mutations involving the voltage-gated sodium channel alpha (I) gene *SCN1A* [3]. This case report presents a detailed account of a 11-year-old patient diagnosed with Dravet syndrome, highlighting the clinical features, diagnostic challenges, treatment strategies, and long-term outcomes.

## 2. Case Presentation

The patient, a female, who is currently 11 years old, was born at term by LSCS to nonconsanguineous parents. She was originally transferred to the Pediatric Department of Sheikh Khalifa Medical City at the age of two with a history of recurrent seizures from the age of four months. She received her first vaccination at the age of four months, after which she developed a high-level fever and left side tonic-clonic convulsions lasting about 15 minutes. She was also admitted twice at the age of ten to the Pediatric Intensive Care Unit with epileptic status. The child had developmental delays including speech and language delay and learning difficulties. A neurological examination of her lower and upper limbs exhibited normal tone and tendon reflexes. In addition, the Babinski sign was normal.

## 3. Diagnostic Evaluation

The diagnostic workup included a comprehensive assessment to rule out other causes of seizures. Electroencephalography (EEG) revealed occasional generalized spike and slow wave discharged more on frontal region, indicating generalized epileptiform activity, consistent with Dravet syndrome. Lumbar puncture done showed no significant findings. An MRI was also done showing mild cerebellar vermis atrophy. In addition, genetic testing was performed, targeting mutations in the *SCN1A* gene, which is associated with most Dravet syndrome cases. In this patients' case, *SCN1A* gene sequencing revealed a heterozygous pathogenic mutation; *c.1344dupT*, *p.Glu449*^*∗*^. Family genetic testing revealed only another sister with the variant gene; however, with milder symptoms while the rest of the family tested negative including the remaining nine siblings.

## 4. Treatment and Management

The patient was started on a combination of antiepileptic drugs, including perampanel and sodium valproate. Other medications that helped with patients' symptoms were clonazepam, baclofen, clonidine, and melatonin.

Alternate treatment modalities including vagal nerve stimulation tried previously did not yield the desired benefit.

Due to the increased risk of sudden unexpected death in epilepsy (SUDEP) associated with Dravet syndrome, close monitoring and seizure precautions were implemented. The patient also received early intervention services to address developmental delays and optimize cognitive functioning.

## 5. Follow-Up and Long-Term Outcomes

Over the course of several years, the patient continued to experience seizures, albeit with varying frequency and intensity. Despite ongoing seizures, a multidisciplinary approach involving neurologists, developmental specialists, and physical therapists has helped manage the patient's symptoms and support their overall well-being. The family receives ongoing education and counseling regarding seizure management, safety measures, and the potential impact on the child's development.

## 6. Discussion

Dravet syndrome is a rare disorder affecting 1 in 15,700 live births [4]. With Dravet syndrome being commonly misdiagnosed and not commonly heard of, it is quite challenging to diagnose and start treatment early for these children.

Studies in the Arab world in general for Dravet syndrome are limited. In Lebanon, a study carried out on 8 patients showed similarity with our patient with regards to seizure presentation, age at first presentation, and the absence of a family history of seizures [5]. Another case report on Dravet syndrome conducted in Saudi Arabia revealed no remarkable radiological findings [6]. However, our patient had mild cerebellar vermis atrophy in the MRI. A case report on two Palestinian siblings confirmed parental mosaicism in the transmission of DS, thereby expanding the spectrum of known *SCN1A* mutations [7]. The possibility of mosaicism was also raised in a study carried out in the United Arab Emirates on three affected male siblings [8] which could also be a factor with our patient since family testing yielded negative results in our case also.

In this case report, the two-year-old child had presented with recurrent seizures since she received vaccination and developed fever at the age of four months. The seizure would last 15 minutes. She was otherwise developmentally normal but was noticed to regress after the seizure episodes.

Seizure presentation may vary with most patients having their first seizure between the ages of five to eight months due to triggers such as fever, vaccination, or absence of any trigger [1]. Seizure type can be focal or generalized tonic-clonic type [1]. This is consistent with our patient described above, although her first seizure occurred at 4 months.

Genetic testing was recommended which revealed the *SCN1A* gene consistent with Dravet syndrome. In addition, her sister was also found to have the variant gene.

Although in about 85% of patients, a mutation of the SCN1A gene is present [9], other genes, such as but not limited to *SCN1B* [10], GABRA1 [11], STXBP1 [11], and *HCN1* [12], have also been implicated in Dravet syndrome although with atypical presentations [13].

The differential diagnosis of DS includes febrile seizures, severe infantile multifocal epilepsy (SIMFE), progressive myoclonic epilepsy (PME), and Lennox–Gastaut syndrome (LGS). Based on clinical features and laboratory investigations, the above conditions were ruled out.

As per the Dravet Syndrome Foundation [14], clinical diagnostic criteria include at least 4 of the following: (a) normal or near-normal cognitive and motor development before seizure onset, (b) two or more seizures with or without fever before 1 year, (c) seizure history consisting of myoclonic, hemiclonic, or generalized tonic-clonic seizures, (d) two or more seizures lasting longer than 10 minutes, and (e) failure to respond to first-line antiepileptic drug therapy with continued seizures after 2 years of age.

Our patient fulfilled all the above clinical criteria for Dravet syndrome.

Although EEG changes can vary from onset to evolution of Dravet syndrome, during wakefulness, background activity is shown to be normal in 50% of the cases, while in the remaining cases, the EEG becomes slow and poorly organised [15] which was seen in our patient. In addition, brain neuroimaging findings on MRI such as focal brain atrophy, cortical dysplasia, and hippocampal sclerosis are found in minority of patients with Dravet syndrome [16]. In our patient, mild cerebellar vermis atrophy was seen in the MRI.

Despite treatment challenges and most patients presenting after a vaccination, immunization should not be withheld in patients with Dravet syndrome. Refractory seizures along with cognitive, motor, and behavioral impairments show that there is a considerable need for newer treatments to target the unmet needs of this condition.

Early diagnosis is pertinent to avoid treatments that are contraindicated and exacerbate seizures [17]. Initial treatments include valproate (VPA) and clobazam (CLB), although they are generally insufficient to control seizures [18]. Stiripentol, cannabidiol, and fenfluramine are the newer treatments proposed for DS [19]. Nonpharmacological treatment modalities such as cannabidiol have also shown to significantly reduce seizures compared to placebo [20]. Another meta-analysis showed ketogenic diet as a safe treatment option for patients with Dravet syndrome [21].

## 7. Conclusion

Dravet syndrome is a challenging disorder that requires a multidisciplinary approach to optimize the management and quality of life for affected individuals. Early diagnosis, genetic testing, and appropriate treatment interventions play crucial roles in mitigating the impact of seizures and associated developmental delays. Continued research and improved access to specialized therapies are essential in enhancing outcomes and providing support to individuals and families affected by Dravet syndrome.

## Data Availability

The data that support the findings of this study are openly available.
